# Telework and Mental Health during COVID-19

**DOI:** 10.3390/ijerph19052602

**Published:** 2022-02-24

**Authors:** Inês Mendonça, Franz Coelho, Paulo Ferrajão, Ana Maria Abreu

**Affiliations:** 1 Institute of Health Sciences, Universidade Católica Portuguesa, 1649-023 Lisbon, Portugal; franzgrc@hotmail.com; 2 Faculty of Social Sciences and Technology, Universidade Europeia, 1500-210 Lisbon, Portugal; paulo.ferrajao@universidadeeuropeia.pt or; 3 Center for Interdisciplinary Research in Health, Universidade Católica Portuguesa, 1649-023 Lisbon, Portugal

**Keywords:** telework, ICTs, mental health, wellbeing, COVID-19

## Abstract

COVID-19 has come to change societal organization. Due to lockdowns, work typologies have been rethought and telework has gained strength. However, the impact of the constant use of information and communication technologies on the mental health of workers needs to be considered. We aimed to investigate the impact of different work conditions on mental health, to which end we disseminated an online questionnaire during lockdowns to assess imagined surveillance, mobile maintenance expectation, communication overload, feelings of entrapment, depression, anxiety, stress, and flourishing in four groups (employed in telework, employed on-site, employed in layoff, and unemployed). We computed mean comparisons and serial mediations. We show that depression and anxiety were more prevalent in women; parents flourished more than people without children; and people with a higher level of education feel more entrapment. Crucially, we show that telework was associated with imagined surveillance and communication overload, which mediated the association with mobile maintenance expectations and entrapment (which was exacerbated by parenthood), impacting mental health and the quality of life. However, this was also partially observed in the remaining work conditions. Finally, flourishing worked as a protector against mental health issues in all work conditions. We discuss this given the massification of digital migration.

## 1. Introduction

Considering the current pandemic situation, resulting from the dissemination of the disease caused by SARS-CoV-2 (COVID-19), one essential social change that was implemented to preserve the economy, despite the need for social isolation, was telework. However, the impact of telework (influenced by the dissemination of information and communication technologies (ICTs)) in our mental health and quality of life is still not known. Currently, it is difficult to conceive of the world without ICTs, which are used for teleworking. These technologies, which include the mobile phone/smartphone, are used by most of us, with the aim of reducing the social space by accessing the internet, and, in particular, by accessing social networks [1]. The use of these technologies has shown several advantages, such as greater work performance and learning [2], cognition [3], and immediate and nonstop remote communication [4,5].

Even before the outbreak of COVID-19, the virtual work regimen had been increasingly adopted around the world, due to the recognition of benefits to those developing their professional activity from home: a more flexible regimen and the promotion of greater worker autonomy [6,7,8]. Telework consists of having a remote work location and the use of ICTs to work [9]. Based on the literature, there are three main categories of telework: regular home-based telework (work from home regularly through ICTs); high mobile telework (work with mobility in several places regularly through ICTs) and occasional telework (work in one or more places outside workplace occasionally) [10]. For this investigation, we focused on studying the regular home-based work condition, as it was adopted as a prevention work condition of COVID-19 [10].

ICT users in teleworking seemed to show certain personality characteristics, such as a sense of responsibility, autonomy, and control over their work, which lead to success [6,8]. Indeed, and according to Tavares [8], the teleworking regimen brings health benefits, mainly because it causes less stress. Delanoeije and Verbruggen [11], for example, report a decrease in general stress levels after the start of teleworking activity, when compared with the previous situation, i.e., working on-site, and when comparing days in telework with days working on-site, in mixed working arrangements. Others have described a greater balance between work and family life [8], which can increase positive emotions such as happiness or joy, depending on individual differences [12].

On the other hand, some health problems associated with ICTs and this type of work regimen have also been described [8]. Gradisar and collaborators [13], Rosen and colleagues [14], and Upreti and Musalay [15], suggested that the excessive use of ICTs may have implications for well-being and mental health, generating anxiety or depression, a propensity for action in multitasking, sleep problems at night, among other symptoms. The sheer speed of information processing calls for speed of response, which might have a strong impact on the mental health [16]. Moreover, since telework imposes on family life, it forces an adaptation and availability that interferes with family routines and habits (e.g., [16]). ICT allows us to be in constant contact with friends and family as well as co-workers and supervisors. This permanent contact, supported by internet connections, can be made anytime and anywhere, invading time beyond working hours and penetrating our personal life [17].

In addition, the regular use of ICTs, namely smartphones, causes a negative impact in terms of cognitive abilities, such as thinking, memory, attention (with implications in the focus of attention, and sustained and divided attention), and emotional regulation [3]. Harmful uses of ICTs have also been described, such as: access to certain apps, multitasking approaches, and notifications. Together, such uses suggest cognitive skill interference [18] and may cause a fear of missing out (FoMO), which is the fear of missing out on information or events by not being connected [15]. FoMO has been referred to as a negative factor, affecting well-being and happiness. The cost of “on the go productivity” has led to distinct patterns of involvement with ICTs, namely smartphones. One that relates to an internal struggle with the habit of being connected, and another that refers to an external obligation to satisfy social expectations of response [19]. According to the authors, the pressure of being connected is leading to a new behavior, i.e., voluntary disconnect. Those who consciously tried to change the way they used their mobile phone, imposed limits on visualization and often chose to leave it behind, or were not available to those who contacted them, although feeling some possible frustration for not getting an immediate response, felt a greater control of their lives and had feelings of happiness and less stress, which is termed the joy of missing out (JoMO) [20].

Connections through social media usage may result in a feeling of imagined surveillance, which provides a sense of being watched all the time [21]. This imagined surveillance guides the personal perception of the social world, impacting how individuals present themselves, expose their lives and engage with ICTs to interact with people [21]. When the demands from ICT channels are high, it may cause a communication overload, as the user needs to handle a lot of different media platforms and technologies to interact with people, causing exhaustion of having to be in touch with others [22,23]. Between the different ICTs, the frequent recourse to smartphones to interact with people also leads to an expectation of relational maintenance, i.e., a mobile- maintenance expectation, which establishes a dependence on technology to feel satisfaction and connectedness with partners [24]. When the mobile maintenance expectation is high, it leads to an overdependence and a sense of entrapment, i.e., being under pressure to be in touch with others and feeling guilty about delays, which decreases the relational satisfaction [24,25]. The overdependence and entrapment feeling associated to the intensive use of ICTs, together with the FoMO, cause digital stress, which is linked to negative mental health outcomes and a compromised quality of life [26]. Even though all these concepts seem to be associated, there is a dearth of direct research on the association between imagined surveillance, communication overload, mobile maintenance expectation, entrapment, mental health and quality of life.

As described above, studies have shown that ICTs have been used even before COVID-19 and that they may have both positive and negative effects on routines and mental health. However, the COVID-19 pandemic changed the way we are living and has demanded an intense adoption of those technologies to work under a context of pressure and social isolation [27], which might directly impact the mental health and quality of life of teleworkers.

Although Portugal was one of the first countries in Europe to implement a regulated virtual work regimen, there are currently significant legislative difficulties for this type of work associated to the limits of labor dependency and the autonomy of the job [28]. In a study carried out by Tavares and collaborators [29], those who transitioned to telework in Portugal, due to the COVID-19 pandemic, referred to a feeling of working harder and identified distractors to their work activity, such as carrying out household chores, taking care of children and social networking. Moreover, these teleworkers reported difficulties, such as the lack of interaction with colleagues, lack of labor resources and managing family life with work schedules. In terms of health and well-being, handicaps were also described by those who worked remotely, such as the absence of a feeling of belonging, isolation, loneliness, lack of interaction and sharing with peers, weak separation between family life and labor [6].

The COVID-19 pandemic changed the routines of workers and intensified the usage of ICTs. However, it also disseminated several other work situations: unemployment, layoff, and the traditional on-site work (with a higher risk of being exposed to the virus). It is possible that telework might impact mental health and the quality of life of workers, as shown before, but the strain of unemployment and layoff [30], as well as the stress of maintaining the usual on-site work during COVID-19 exposure fear [31], could impact mental health just as well. Notwithstanding, if we consider that teleworking is supported by the use of ICTs [27], and that excessive use of ICTs can impact mental health and the quality of life [17], we expect that those who stay at home in telework will feel more anxious [32] and will feel more burdened with work driven by the FoMO phenomenon [33]. Having said that, our main objective with this investigation was to analyze different work situations and the psychological experiences in times of social isolation, considering the impact that the excessive use of ICTs (associated to social isolation and telework) might generate on mental health and quality of life.

Here, we aimed to examine how the different work conditions (telework, unemployment, layoff, and on-site work) and different sociodemographic characteristics (gender, parenting and education) impact the psychological experiences from use of ICTs (imagined surveillance, mobile maintenance expectation, communication overload and entrapment), mental health (depression, anxiety, and stress) and quality of life (flourishing). To the best of our knowledge, there is no previous study that examined the association of these variables in these different work conditions. This investigation aimed to analyze the levels of mobile maintenance, entrapment, mental health (depression, anxiety and stress) and quality of life (flourishing) through the mediation of both imagined surveillance and communication overload in different settings of work conditions. In addition, we aimed to analyze the differences between these variables in distinct sociodemographic characteristics. Having said that, we posited the following hypotheses:

**Hypothesis** **1** **(H1).** *High mobile maintenance expectation is related to high entrapment.*

**Hypothesis** **2** **(H2).** *High mobile maintenance expectation and entrapment should be associated with increased depression, anxiety, and stress.*

**Hypothesis** **3** **(H3).** *High levels of depression, anxiety and stress would be correlated with lower levels of flourishing.*

**Hypothesis** **4** **(H4).** *Teleworkers might present higher mobile maintenance expectation and entrapment, through the mediation of imagined surveillance and communication overload, which would increase levels of depression, anxiety and stress and decrease flourishing.*

**Hypothesis** **5** **(H5).** *On-site workers, unemployed persons and layoff workers might also have low levels of flourishing and high levels of depression, anxiety, and stress, but not correlated to mobile maintenance expectation and entrapment, through the mediation of imagined surveillance and communication overload, as they are more exposed to the COVID-19 virus but depend less on ICTs.*

**Hypothesis** **6** **(H6).** *Gender, parenting and education should differently impact all those variables.*

## 2. Materials and Methods

### 2.1. Measures

We developed a questionnaire, applied online, concerning the psychological experiences in times of lockdown. This questionnaire was composed of the Portuguese version of the Anxiety, Depression and Stress Scale—21 items [34]; the Portuguese version of the Flourishing Scale [35]; and the adapted and translated version of the Scale of Expectation of Permanence on Mobile and Entrapment [24] to Portuguese.

Considering the impact that telework can have on mental health, namely in terms of the development of states of anxiety, depression, and stress, it seemed relevant to apply the short version of the Depression, Anxiety, and Stress Scale (DASS-21) [36]. The DASS-21 scale is a self-report instrument to measures three related negative emotional states, i.e., depression, anxiety and stress or tension. The DASS-21 consists in 3 subscales, with 7 items each, measured on a four-point Likert scale from 0 to 3, wherein 0 corresponds to “totally disagree” and 3 “totally agree”. An example of an item of this scale would be “I had difficulty calming down”. High Scores reveal a person with high levels of anxiety, depression and/or stress. In our study, we used the Portuguese version of the DASS-21, adapted by Pais-Ribeiro, Honrado, & Leal [34].

We also used the Portuguese version [35] of the Flourishing Scale [37]. The Flourishing scale is a brief 8-item scale, used as a measure of individual self-perception of success in relationships, optimism, purpose, and self-esteem. Respondents answered a 7-point Likert scale from 1 (strongly disagree) to 7 (strongly agree), wherein high scores revealed a person with many psychological resources and strengths. An example of an item of this scale would be “I live a purposeful and meaningful life”.

Further, mobile phone use can affect expectations of permanence, dependence experience, overdependence, and entrapment, as well as interfere and affect the satisfaction of close friendship relationships [24]. In order to better understand the expectations of the participants regarding their mobile phone maintenance, as well as the pressure/stress to contact or be contacted by co-workers or superiors (Entrapment), we applied two scales developed by Hall & Baym [24]: Mobile Maintenance Expectations, composed by 9 items, measured on a Likert scale from 1 to 5, wherein 1 corresponds to ‘never’ and 5 corresponds to ‘always’. An example of an item of this scale would be “My superiors/colleagues expect me to call/text to speak about the things we are planning to do together”; and the Entrapment scale, consisting of 7 items, measured on a Likert scale from 1 to 5, wherein 1 corresponds to ‘Strongly Disagree’ and 5 corresponds to Strongly Agree. An example of an item of this scale would be “Sometimes I don’t want my superiors/colleagues to contact me”. We translated, backtranslated and adapted these scales to the reality of telecommuting.

In addition to the scales, participants were also asked to answer two questions. The first being: “Do you feel pressured to place your superiors/colleagues on social networks that you would rather keep private?”. With this question we aimed to investigate the putative feeling of being watched by co-workers, i.e., work imagined surveillance. The second question was: “Do you feel pressured to have more than one platform (i.e., Email, WhatsApp, Telephone, Teams, Zoom, Skype, etc …)?” With this question, we aimed to investigate the work communication overload caused by the high demand of ICTs channels. Participants were also required to provide some sociodemographic data, such as: nationality; gender; district of permanent residence; age; marital status; number of children or other dependents (how many and age); schooling situation; work situation at the time of the pandemic; profession; and work regimen.

### 2.2. Participants and Procedure

A sample of 500 participants took part in this study that took place during the first COVID-19 pandemic lockdown in Portugal (data was collected from April to July 2020). Sample characteristics are presented in Table 1. The mean age of the sample was 44.6 years (SD = 9.6; range 20 to 75 years); most of the participants were women (81.8%). More than half of the participants had a bachelor’s degree; nearly a quarter of the participants had a master’s degree. Regarding marital status, more than half of the participants were married or cohabiting; nearly one quarter of the participants were single. Most participants (70.0%) had children; more than half the participants had one child or two children. Almost all participants were Portuguese. Most participants were working on behalf of others and most of the participants were working from home (telework) during the lockdown.

We prepared an electronic version of a questionnaire about the psychological experiences in times of lockdown, as there was contact restriction imposed for pandemic control. The link to the research site was shared online, through various social media platforms, such as Facebook, Instagram, and WhatsApp, following a snowball sampling strategy to recruit a large group of respondents varying in terms of socio-demographic characteristics. The participants were able to access the link and complete the questionnaire that took just under 10 min to complete. All participants volunteered to take part in the study and did not receive any monetary reward.

Inclusion criteria related to demographic characteristics were: being older than 20 years of age and becoming unemployed, working in layoff, teleworking, or maintaining on-site work during the COVID-19 pandemic. We recruited only participants 20 years or older since we assumed that, at this age, participants had already started their working life and may have already undergone other work situations, such as unemployment and/or layoff. The participants were informed about the anonymity of the study and the study was approved by the local university ethics committee.

### 2.3. Data Analysis

Data analysis was conducted using IBM SPSS Statistics for Windows (version 27, IBM, New York, NY, USA). Data were first analyzed for correlation matrix on all variables. Multiple Pearson correlation analyses were conducted to test bi-variate relationships between the study variables. Previous literature considered that if a coefficient value lies between ±0.50 and ±1, then it is said to be a strong correlation; if it lies between ±0.30 and ±0.49, then it is said to be a medium correlation; and if it lies below 0.29, then it is said to be a small correlation [38].

We conducted a one-way between-groups multivariate analysis of variance (MANOVA) to compare multivariate group means of professional situation (telework, on-site work and not working) on levels of entrapment, flourishing, mobile maintenance expectation, depression, anxiety, and stress. Data from unemployed participants and lay-off participants (not working) were analyzed together due to the small size of the group of unemployed participants. Bonferroni correction was used to counteract multiple-pairwise comparisons. Preliminary assumption testing was conducted to check for normality and homogeneity of variance.

We conducted a two-way multivariate analysis of variance (two-way MANOVA) to analyze the interaction between the professional situation with the sample characteristics (gender, parental status, education, imagined surveillance, and communication overload) on levels of entrapment, flourishing, mobile maintenance expectation, depression, anxiety, and stress. Bonferroni correction was used to counteract multiple-pairwise comparisons. Preliminary assumption testing was conducted to check for multivariate normality and homogeneity of variance-covariance matrices.

To test our hypotheses, we conducted serial mediation models using a multiple step mediation methodology, with a bootstrapped confidence interval for indirect effects (Model 6) [39]. The final model included three outcome variables (depression, anxiety, and stress symptoms), so we chose to examine this model through structural equation modelling (SEM). Specifically, the following was examined: (a) if imagined surveillance and communication overload were directly linked to depression, anxiety, and stress symptoms; (b) if imagined surveillance and communication overload were indirectly linked to depression, anxiety, and stress symptoms through entrapment, mobile maintenance expectations and flourishing; (c) if a two-step mediation process exists in which imagined surveillance and communication overload were indirectly linked to depression, anxiety, and stress symptoms through entrapment, mobile maintenance expectations and flourishing. These models were independently tested on three groups of participants: participants who were working from home (telework group), participants who were working in their usual workplace (on-site work group), and participants who were in layoff or unemployed (not-working group). Data from unemployed participants and lay-off participants were analyzed together due to the small size of the group of unemployed participants.

Finally, to test the serial mediation model, we employed an SEM strategy [40] using AMOS software (Version 26, IBM, NY, USA) [41] and employing the ML method. We adopted the following criteria for SEM models fit: (a) a χ2 test, (b) the root mean square error of approximation (RMSEA), (c) the comparative fit index (CFI), (d) the normed fit index (NFI), (e) Tucker Lewis index (TLI), and (f) standardized root mean square residual (SRMR). In addition, we adopted the following criteria for each model fit: chi-square value should be non-significant, CFI, TLI and NFI > 0.95, and the RMSEA and SRMR should range from 0.00 to 0.08. We adopted Preacher and Hayes’ procedures [42] to assess significance of indirect paths, a bootstrapped confidence interval for the ab indirect effect. A total of 5000 bootstrapped samples were obtained to estimate indirect effects of each mediator. We computed bias corrected, accelerated 95% confidence intervals (CIs) to measure statistical significance for each mediator’s “ab” paths and the two-step mediation. A confidence interval that does not include zero reflects evidence of a significant indirect effect or significant mediation.

## 3. Results

### 3.1. Correlations

As we can see in Table 2, entrapment showed a moderate positive association with mobile maintenance expectation, depression, anxiety, and stress levels, and a weak negative association with flourishing. Flourishing presented a moderate negative correlation with depression, and moderate to weak correlations with both anxiety and stress. The association of mobile maintenance expectation with all psychological symptoms’ levels were weakly positive. All psychological symptoms’ levels were positively and strongly linked with each other. The remaining associations were all nonsignificant.

### 3.2. Mean Comparisons

We conducted a MANOVA to compare multivariate group means for levels of entrapment, flourishing, mobile maintenance expectation, depression, anxiety, and stress. The independent variable was work situation. We compared the following groups: telework group, on-site work group, and not-working group. As mentioned above, considering the reduced number of unemployed participants, these participants were collapsed together with participants who were not working during lockdown (participants in layoff) in subsequent analyses. We conducted preliminary assumption testing to check for normality, linearity, univariate and multivariate outliers, homogeneity of variance-covariance matrices, and multicollinearity, with no serious violations noted. There were statistically significant differences between the different work situations on the combined dependent variables, F(12, 984) = 2.66, *p* = 0.002; Wilks’ Lambda = 0.94; partial eta squared = 0.03. As we can see in Table 3, when the results for the dependent variables were considered separately, using a Bonferroni adjusted alpha level of 0.05, we found a statistically significant difference between the different work situations only on the levels of Entrapment. A Bonferroni post-hoc test was used to perform pairwise comparisons between group means, but controlling the overall error rate by setting the error rate for each test to the experiment wise error rate divided by the total number of tests. Results showed that, for the entrapment scores, there were statistically mean differences between the telework group and the not-working group (n = 75, *p* = *0*.001; CI mean differences: 0.16; 0.76) and the on-site work group (n = 55, *p* = *0*.002; CI mean differences: 0.14; 0.83). The remaining comparisons were all nonsignificant. The telework group showed higher scores on Entrapment compared to the other two groups.

We conducted a two-way MANOVA to analyze the interaction between professional situation and gender on levels of entrapment, flourishing, mobile maintenance expectation, depression, anxiety, and stress. Preliminary assumption testing was conducted with no serious violations noted. We observed a statistically significant interaction effect between gender and professional situation on the combined dependent variables, F(12, 978) = 1.03, *p* = 0.61; Wilks’ Λ = 0.975. We also observed between-subjects differences for gender on levels of depression (F(1) = 4.92, *p* < 0.05), anxiety (F(1) = 13.25, *p* < 0.001), and stress (F(1) = 8.81, *p* < 0.001). Women showed higher levels on both depression (n = 409, M = 0.57, SD = 0.60) and anxiety (n = 409, M = 0.52, SD = 0.59) compared to men (depression: n = 91, M = 0.36, SD = 0.43: anxiety: n = 409, M = 0.15, SD = 0.35).

We conducted a two-way MANOVA to analyze the interaction between professional situation and having, or not, a child on levels of entrapment, flourishing, mobile maintenance expectation, depression, anxiety, and stress. Preliminary assumption testing was conducted with no serious violations noted. We did not observe a statistically significant interaction effect between having or not having a child and professional situation on the combined dependent variables, F(12, 978) = 0.72, *p* = 0.73; Wilks’ Λ = 0.983. We also observed between-subjects differences between parents and non-parents on levels of flourishing (F(1) =10.91, *p* < 0.001). Participants who had a child presented higher levels of flourishing (n = 350, M = 5.82, SD = 0.88) compared with participants who did not have a child. (n = 150, M = 5.55, SD = 1.07).

We conducted a two-way MANOVA to analyze the interaction between professional situation and education on levels of entrapment, flourishing, mobile maintenance expectation, depression, anxiety, and stress. Preliminary assumption testing was conducted with no serious violations noted. We did not observe a statistically significant interaction effect between education and professional situation on the combined dependent variables, F(24, 1692) = 1.30, *p* = 0.15; Wilks’ Λ = 0.938. Moreover, we observed between-subjects differences for education on levels of entrapment (F(2) = 6.12, *p* < *0*.01). Participants with twelve or fewer years of education presented lower levels of entrapment compared to other participants.

**Figure 1:**
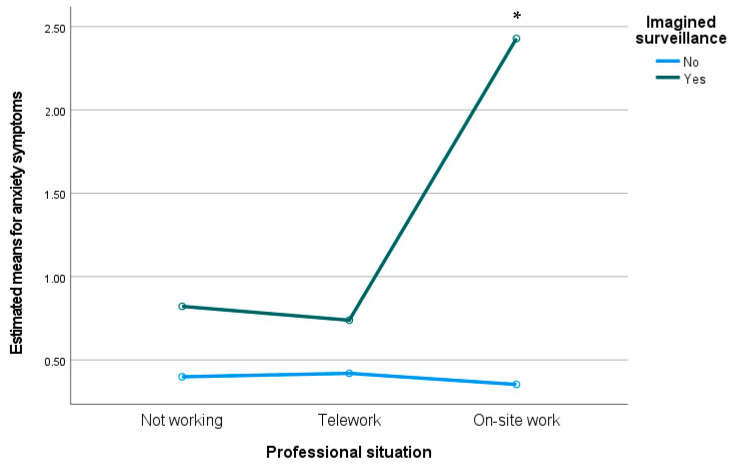
Estimated means for anxiety symptoms through the interaction between professional situation and imagined surveillance. * *p* < 0.05.

**Figure 2:**
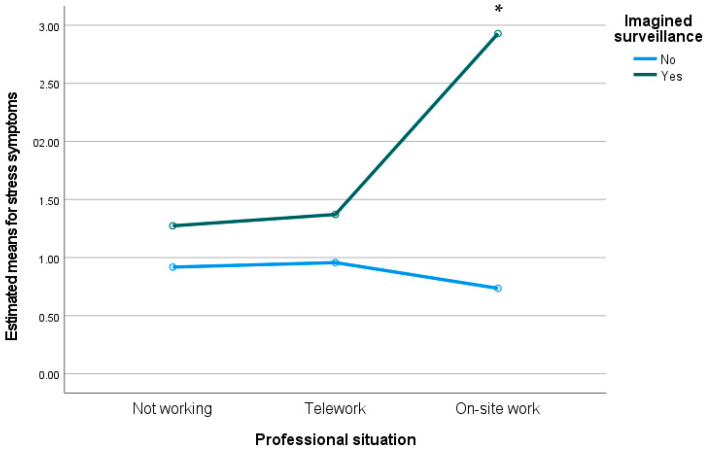
Estimated means for stress symptoms through the interaction between professional situation and imagined surveillance. * *p* < 0.05.

We conducted a two-way MANOVA to analyze the interaction between professional situation and imagined surveillance on levels of entrapment, flourishing, mobile maintenance expectation, depression, anxiety, and stress. Preliminary assumption testing was conducted, with no serious violations noted. We observed a statistically significant interaction effect between imagined surveillance and professional situation on the combined dependent variables, F(12, 978) = 3.36, *p* < 0.001; Wilks’ Λ = 0.922. Moreover, we observed between-subjects differences on levels of anxiety (F(2) = 9.85, *p* < 0.001) and stress (F(2) = 5.96, *p* < 0.01). As can be seen in Figure 1 and Figure 2, participants who were working on-site and who appraised imagined surveillance presented higher levels on both anxiety and stress.

**Figure 3:**
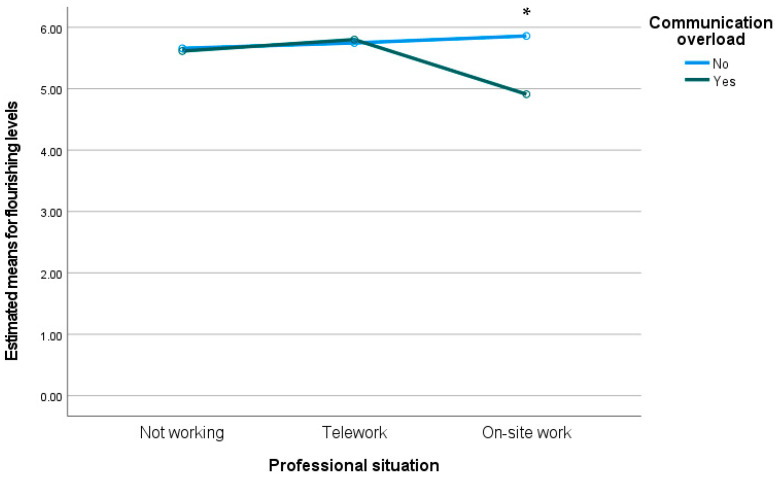
Estimated means for flourishing levels through the interaction between professional situation and communication overload. * *p* < 0.05.

**Figure 4:**
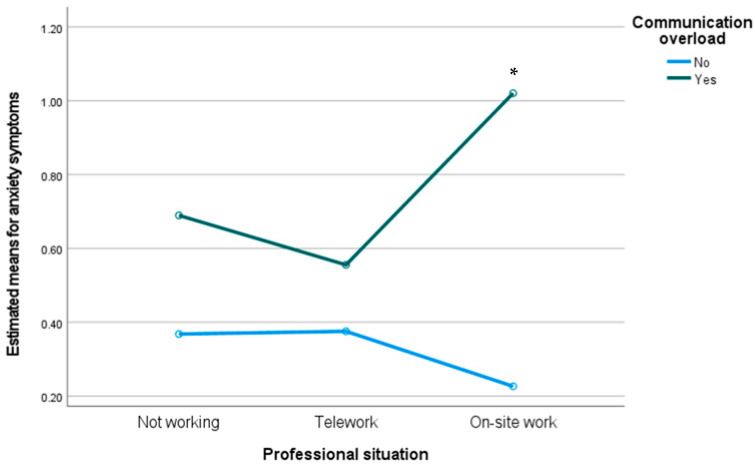
Estimated means for anxiety symptoms through the interaction between professional situation and communication overload. * *p* < 0.05.

We conducted a two-way MANOVA to analyze the interaction between professional situation and communication overload on levels of entrapment, flourishing, mobile maintenance expectation, depression, anxiety, and stress. Preliminary assumption testing was conducted with no serious violations noted. We observed a statistically significant interaction effect between communication overload and professional situation on the combined dependent variables, F(12, 978) = 2.24, *p* < 0.01; Wilks’ Λ = 0.943. Moreover, we observed between-subjects differences on levels of flourishing (F(2) = 5.36, *p* < *0*.01) and anxiety (F(2) = 6.01, *p* < *0*.01). As we can see in Figure 3 and Figure 4, participants who were working on-site who reported communication overload presented lower levels of flourishing and higher levels of anxiety compared to the other groups.

### 3.3. Analysis of Serial Mediation

As we mentioned above, models were independently tested on the telework group, the on-site work group and the not-working group. We conducted serial mediation models using a multiple step mediation methodology on the telework group.

#### 3.3.1. Telework Group

First, we examined the existence of significant direct relations between imagined surveillance and communication overload to depression, anxiety, and stress symptoms. This model poorly fits the observed data (χ2 (1) = 45.45, *p* < 0.01; NFI = 0.95; CFI = 0.95; TLI = 0.86; RMSEA = 0.15; SMSR = 0.09). Imagined surveillance was significantly associated with higher levels of depression (b = 0.31, *p* < 0.01, 95% CI, 0.15, 0.45), anxiety (b = 0.27, *p* < 0.01, 95% CI, 0.12, 0.42), and stress symptoms (b = 0.28, *p* < 0.01, 95% CI, 0.09, 0.47). Communication overload was significantly associated with higher levels of both depression (b = 0.16, *p* < 0.01, 95% CI, 0.05, 0.27) and stress symptoms (b = 0.30, *p* < 0.01, 95% CI, 0.15, 0.45), but it was not associated with anxiety symptoms (b = 0.11, *p* = 0.06, 95% CI, −0.01, 0.23).

Subsequently, we examined whether one step mediators—mobile maintenance expectations, entrapment, and flourishing—had direct paths to depression, anxiety, and stress symptoms. This model fit the observed data well (χ2 (2) = 6.93, *p* < 0.05; NFI = 0.96; CFI = 0.97; TLI = 0.89; RMSEA = 0.07; SMSR = 0.07). The direct paths from mobile maintenance expectations to depression (b = 0.09, *p* = 0.33, 95% CI, −0.09, 0.27), anxiety (b = 0.17, *p* = 0.10, 95% CI, −0.03, 0.37), and stress symptoms (b = 0.19, *p* = 0.08, 95% CI, −0.02, 0.40) were non-significant. Entrapment was significantly associated with higher levels of depression (b = 0.21, *p* < 0.01, 95% CI, 0.05, 0.37), anxiety (b = 0.27, *p* < 0.01, 95% CI, 0.12, 0.42), and stress symptoms (b = 0.43, *p* < 0.01, 95% CI, 0.27, 0.59). Flourishing was significantly associated with lower levels of depression (b = −0.18, *p* < 0.01, 95% CI, −0.29, −0.07) and anxiety symptoms (b = −0.19, *p* < 0.01, 95% CI, −0.29, −0.09), but it was not associated with stress symptoms (b = −0.10, *p* = 0.09, 95% CI, −0.22, 0.02).

Finally, we specified a model in which imagined surveillance and communication overload had direct paths to depression, anxiety, and stress symptoms, as well as indirect paths through mobile maintenance expectations, entrapment, and flourishing (one-step mediation). The mediation model fit the observed data well (χ2 (2) = 4.34, *p* = 0.11; NFI = 1.0; CFI =1.0; TLI = 0.97; RMSEA = 0.05; SMSR = 0.02). The direct paths from imagined surveillance to both depression (b = 0.18, *p* < 0.05, 95% CI, 0.03, 0.33) and anxiety symptoms (b = 0.16, *p* < 0.05, 95% CI, 0.01, 0.31) remained significant when one step mediators were included in the model. However, the direct paths from imagined surveillance to stress symptoms (b = 0.11, *p* = 0.30, 95% CI, −0.10, 0.32) were no longer significant when one step mediators were included in the model. Nevertheless, when applying the bootstrapping analysis, total indirect effect through entrapment to depression (b = 0.12, *p* < 0.001, 95% CI, 0.05, 0.19), anxiety (b = 0.12, *p* < 0.001, 95% CI, 0.05, 0.19), and stress symptoms (b = 0.18, *p* < 0.001, 95% CI, 0.09, 0.27) were significant. The total indirect effect through mobile maintenance expectations to anxiety (b = 0.09, *p* < 0.05, 95% CI, 0.01, 0.17) were significant, but they were neither significant to depression (b = 0.08, *p* = 0.07, 95% CI, −0.01, 0.17) nor stress symptoms (b = −0.01, *p* = 0.99, 95% CI, −0.13, 0.11). The total indirect effect through flourishing to all the variables was non-significant.

The direct paths from communication overload to stress symptoms (b = 0.16, *p* < 0.05, 95% CI, 0.01, 0.31) remained significant when one step mediators were included in the model. However, the direct paths from communication overload to both depression (b = 0.09, *p* = 0.15, 95% CI, −0.03, 0.21) and anxiety symptoms (b = 0.04, *p* = 0.56, 95% CI, −0.08, 0.16) were not significant when one step mediators were included in the model. However, when applying the bootstrapping analysis, total indirect effect through entrapment to depression (b = 0.07, *p* < 0.05, 95% CI, 0.01, 0.13), anxiety (b = 0.08, *p* < 0.05, 95% CI, 0.01, 0.15), and stress symptoms (b = 0.12, *p* < 0.05, 95% CI, 0.03, 0.21) were significant. All the remaining indirect effects were non-significant. Specifically, the one-step indirect effects results indicated that imagined surveillance was significantly associated with higher levels of entrapment that was associated with higher levels of depression, anxiety, and stress symptoms.

**Figure 5:**
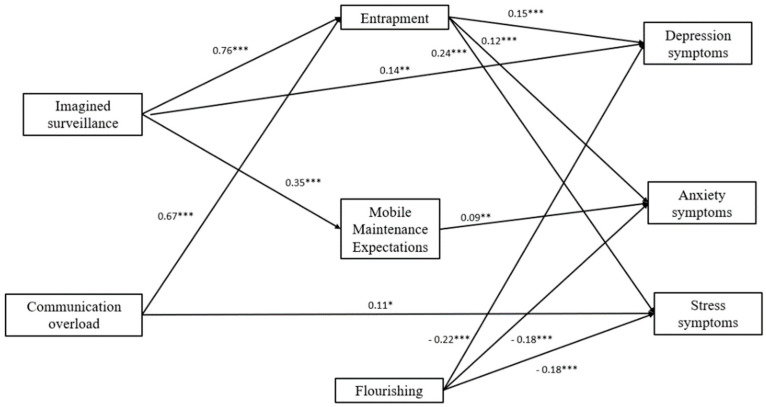
A serial mediational integrated model for depression, anxiety, and stress symptoms by imagined surveillance and communication overload through entrapment, mobile maintenance expectations, and flourishing in the telework group. Rectangles indicate measured variables. Unidirectional arrows depict statistically significant directional links. Standardized maximum likelihood parameters are used. n = 370; * *p* < 0.05, ** *p* < 0.01, *** *p* < 0.001.

Specifically, the one-step indirect effects results indicated that both imagined surveillance and communication overload were significantly associated with higher levels of entrapment that were associated with higher levels of depression, anxiety, and stress symptoms. Likewise, imagined surveillance was significantly associated with higher levels of mobile maintenance expectations, which, in turn, was associated with high levels of anxiety symptoms. Communication overload was directly associated with higher levels of stress symptoms. Lower levels of flourishing were associated with higher levels of depression, anxiety, and stress symptoms. After omitting non-significant paths (i.e., imagined surveillance → flourishing; communication overload → flourishing; mobile maintenance expectations → depression; mobile maintenance expectations → stress) our final model (Figure 5) fit the observed data well (χ^2^ (8) = 9.31, *p* = 0.31; NFI = 0.99; CFI = 1.0; TLI = 1.0; RMSEA = 0.02; SMSR = 0.03).

#### 3.3.2. On-Site Work Group

A similar procedure was conducted on the on-site work group. The model testing the existence of significant direct relations between imagined surveillance and communication overload to depression, anxiety, and stress symptoms showed a poor fit (χ2 (1) = 6.32, *p* < 0.05; NFI= 0.97; CFI = 0.97; TLI = 0.73; RMSEA = 0.11; SMSR = 0.11). Imagined surveillance was significantly associated with higher levels of anxiety (b = 1.64, *p* < 0.01, 95% CI, 0.93, 2.35) and stress symptoms (b = 1.82, *p* < 0.01, 95% CI, 0.95, 2.69), but the direct paths to depression symptoms were non-significant (b = 0.35, *p* = 0.37, 95% CI, −0.40, 1.05). Communication overload was significantly associated with higher levels of both depression (b = 0.58, *p* < 0.01, 95% CI, 0.25, 0.91), anxiety (b = 0.56, *p* < 0.05, 95% CI, 0.25, 0.87), and stress symptoms (b = 0.48, *p* < 0.05, 95% CI, 0.11, 0.85).

The model in which one step mediators had direct paths to depression, anxiety, and stress symptom showed a good fit to the data (χ2 (2) = 2.82, *p* = 0.24; NFI = 0.99; CFI =1.0; TLI = 0.97; RMSEA = 0.08; SMSR = 0.06). Entrapment was significantly associated with higher levels of depression (b = 0.21, *p* < 0.01, 95% CI, 0.08, 0.34), anxiety (b = 0.27, *p* < 0.01, 95% CI, 0.12, 0.42), and stress symptoms (b = 0.43, *p* < 0.01, 95% CI, 0.27, 0.59). Flourishing was significantly associated with lower levels of both depression (b = −0.18, *p* < 0.01, 95% CI, −0.30, −0.06) and anxiety symptoms (b = −0.19, *p* < 0.01, 95% CI, −0.29, −0.09). The remaining associations were non-significant.

Next, the model in which imagined surveillance and communication overload had direct paths to depression, anxiety, and stress symptoms, as well as indirect paths through mobile maintenance expectations, entrapment, and flourishing (one-step mediation) showed a good fit to the data (χ2 (1) = 0.71, *p* = 0.40; NFI = 1.0; CFI =1.0; TLI = 1.0; RMSEA = 0.01; SMSR = 0.02). The direct paths from imagined surveillance to both anxiety (b = 1.57, *p* < 0.01, 95% CI, 0.88, 2.26) and stress symptoms (b = 1.55, *p* < 0.01, 95% CI, 0.77, 2.33) remained significant when one step mediators were included in the model. However, when applying the bootstrapping analysis, total indirect effect through entrapment, mobile maintenance expectations and flourishing to depression, anxiety, and stress symptoms were non-significant. The direct paths from communication overload to depression, anxiety and stress symptoms when one-step indicators were introduced in the model were non-significant. However, when applying the bootstrapping analysis, total indirect effect through entrapment to depression (b = 0.16, *p* < 0.05, 95% CI, 0.02, 0.30), anxiety (b = 0.16, *p* < 0.05, 95% CI, 0.03, 0.29), and stress symptoms (b = 0.34, *p* < 0.01, 95% CI, 0.19, 0.49) were significant. Likewise, the total indirect effect through flourishing to depression (b = −0.16, *p* < 0.01, 95% CI, −0.27, −0.05) and anxiety symptoms (b = −0.14, *p* < 0.05, 95% CI, −0.24, −0.04) were significant.

**Figure 6:**
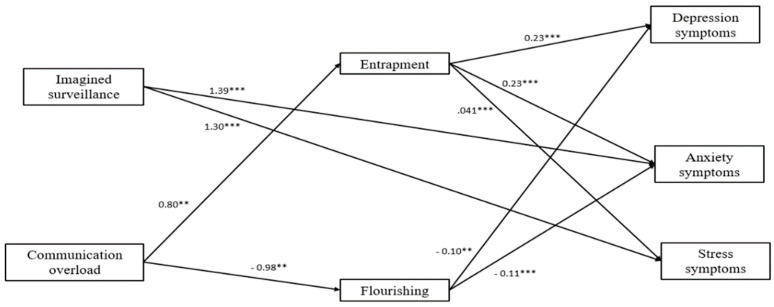
A serial mediational integrated model for depression, anxiety, and stress symptoms by imagined surveillance and communication overload through entrapment, mobile maintenance expectations, and flourishing in the on-site work group. Rectangles indicate measured variables. Unidirectional arrows depict statistically significant directional links. Standardized maximum likelihood parameters are used. n = 55; ** *p* < 0.01, *** *p* < 0.001.

Specifically, the one-step indirect effects results indicated that communication overload was significantly associated with higher levels of entrapment that were associated with higher levels of depression, anxiety, and stress symptoms. Communication overload was associated with lower levels of flourishing, which, in turn, was associated with higher levels of depression and anxiety symptoms. Imagined surveillance was directly associated with higher levels of both anxiety and stress symptoms. After omitting non-significant paths, our final model (Figure 6) fit the observed data well (χ^2^ (13) = 13.97, *p* = 0.38; NFI = 0.95; CFI =1.0; TLI = 1.0; RMSEA = 0.04; SMSR = 0.08).

#### 3.3.3. Not-Working Group

Finally, we conducted serial mediations on the not-working group. As in the two previous groups, the model in which we tested the direct relations between imagined surveillance and communication overload to depression, anxiety, and stress symptoms showed a poor fit to the data (χ2 (1) = 5.22, *p* < 0.05; NFI= 0.97; CFI = 0.97; TLI = 0.82; RMSEA = 0.14; SMSR = 0.08). Imagined surveillance was only significantly associated with higher levels of anxiety (b = 0.34, *p* < 0.01, 95% CI, 0.06, 0.62). Communication overload was significantly associated with higher levels of anxiety (b = 0.25, *p* < 0.05, 95% CI, 0.03, 0.47) and stress symptoms (b = 0.41, *p* < 0.01, 95% CI, 0.11, 0.72).

The model in which one step mediators had direct paths to depression, anxiety, and stress symptoms showed a good fit to the data (χ2 (2) = 2.23, *p* = 0.32; NFI = 0.99; CFI =1.0; TLI = 0.99; RMSEA = 0.05; SMSR = 0.05). Entrapment was only significantly associated with higher levels of anxiety (b = 0.14, *p* < 0.05, 95% CI, 0.03, 0.25). Flourishing was significantly associated with lower levels of depression (b = −0.26, *p* < 0.01, 95% CI, −0.37, −0.15), anxiety (b = −0.14, *p* < 0.01, 95% CI, −0.24, −0.04), and stress symptoms (b = −0.17, *p* < 0.05, 95% CI, −0.31, −0.03). The remaining associations were non-significant.

**Figure 7:**
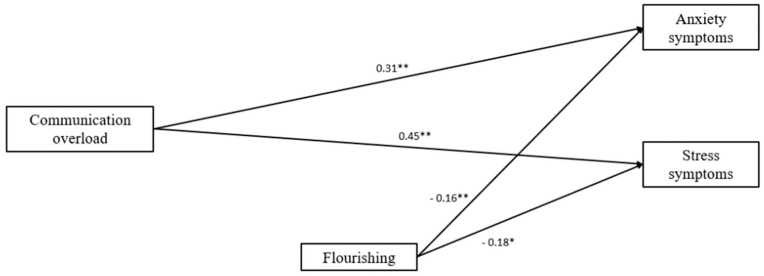
A serial mediational integrated model for depression, anxiety, and stress symptoms by imagined surveillance and communication overload through entrapment, mobile maintenance expectations, and flourishing in the not-working group. Rectangles indicate measured variables. Unidirectional arrows depict statistically significant directional links. Standardized maximum likelihood parameters are used. n = 75; * *p* < 0.05, ** *p* < 0.01.

Finally, the model (Figure 7) in which imagined surveillance and communication overload had direct paths to depression, anxiety, and stress symptoms, as well as indirect paths through mobile maintenance expectations, entrapment, and flourishing (one-step mediation) showed a good fit to the data (χ2 (3) = 3.49, *p* = 0.32; NFI = 0.98; CFI =1.0; TLI = 0.98; RMSEA = 0.05; SMSR = 0.03). There were direct paths from imagined surveillance to depression, anxiety, and stress symptoms when one step mediators were included in the model. When we applied the bootstrapping analysis, and the total indirect effect through entrapment, mobile maintenance expectations and flourishing to depression, anxiety, and stress symptoms was non-significant. The direct paths from communication overload to anxiety (b = 0.25, *p* < 0.05 95% CI, 0.03, 0.47) and stress symptoms (b = 0.44, *p* < 0.01, 95% CI, 0.13, 0.75) when one-step indicators were introduced in the model were significant. Likewise, when applying the bootstrapping analysis, the total indirect effect through one-step mediators to depression, anxiety, and stress symptoms was non-significant. Results indicated that communication overload was directly associated with higher levels of both anxiety and stress symptoms. After omitting non-significant paths, our final model fit the observed data well (χ^2^ (1) = 0.03, *p* = 0.86; NFI = 1.0; CFI =1.0; TLI = 1.0; RMSEA = 0.01; SMSR = 0.01).

## 4. Discussion

The COVID-19 pandemic has changed the way we live, exacerbating the usage of ICTs and transforming our routines. Here, we aimed to answer how the usage of ICTs when working at home, i.e., teleworking, has impacted the mental health and quality of life of workers. In this study, we explored the association between different work conditions with imagined surveillance, communication overload, mobile maintenance expectation, entrapment, flourishing, depression, anxiety, and stress. We evaluated the global correlations between the variables, and we proved our first hypothesis (H1) concerning a possible positive correlation between entrapment and mobile maintenance expectation. We also found a positive correlation between depression, anxiety and stress with high entrapment and high mobile maintenance expectation, proving our second hypothesis (H2). In addition, flourishing presented a negative correlation with depression, anxiety, and stress, in accordance with our third hypothesis (H3). These findings are sustained by the expectation of relational maintenance leading to an overdependence of technology and a sense of being under pressure to be in touch with others, decreasing relational satisfaction [24,25], impacting mental health and compromising quality of life [26]. Our sixth hypothesis (H6) pertained to how sociodemographic data (gender, parenting and education) might differently impact the variables, which we discuss below. Later, we will discuss the other hypothesis.

### 4.1. Sociodemographic Data

#### 4.1.1. Women and Mental Health

We found that, compared to men, women had a higher mean score of depression and anxiety. This is supported by other recent studies wherein it was shown that the lockdown caused by COVID-19 impacted the mental health of people, but that women were significantly more burdened [43]. In another study, Shahriarirad and collaborators [44] investigated the risk factors resulting from the COVID-19 pandemic. The authors found that being female, a younger adult, belonging to a larger household and being a health professional, constituted risk factors for the development of symptoms of depression and anxiety. Other pre-pandemic studies had analyzed the content that women normally published on social media and concluded that their posts tended to be more related to sadness and anxiety than men’s and that women seek more social support, worrying more about their community and family than about themselves [45]. In addition, people who worry more, are more socially isolated and receive less social support, tend to have more mental health problems [46]. Women tend to seek and give more social support through social network sites [47,48], and, even though we did not find any gender differences between entrapment levels, we might assume that women depended more on technology during the lockdown, to provide and seek social support. In addition, women working from home tend to take on more household duties and to help their relatives more, which increases the responsibilities and affects the balance between work and personal life [49]. Together, this literature sustains our own results, mainly during the lockdown, as women might be concerned about social issues, with increased pressure to be in touch with other people, which might potentially impair their mental health.

#### 4.1.2. Parental Status and Flourishing

We showed that people who have children have higher scores in flourishing, compared with people with no children. COVID-19 imposed a lockdown and warranted parents and their children to spend more time at home, making use of ICTs to maintain social communication, work, study and entertain [50]. COVID-19 increased conflicts between work and family, but it did not decrease the quality of perceived parenting, indicating that it is possible for some parents to separate the professional conflicts from issues related to their children [51]. When there are rules and limits to the usage of ICTs in the family, its usage is not harmful [16]. In addition, it is proven that having a child increases the life satisfaction of parents [52] and our results show that being a parent might work as a protector of mental health issues and an enhancer of quality of life. On the other hand, some literature shows the opposite of our finding and say that the use of technology during parenting moments, i.e., technology interfering in family time (technoference) seems to result in worse behavior and development of social competencies in children, which leads to an increase of parent stress [50,53]. The increased time spent on ICTs, together with a more stressful environment at home, caused by technoference during parenting, is in line with our own results of higher entrapment feelings in parents.

#### 4.1.3. Education and Entrapment

People with more years in education training (who have a bachelor’s, MSc or a PhD degree) had higher entrapment scores compared to people with less than twelve years of education. Previous literature has shown that people with higher levels of education (at least a bachelor’s degree) hold more white-collar jobs (i.e., office work) and fewer blue-collar jobs (i.e., manual work), compared to people who do not have at least a bachelor’s degree [54]. In addition, white collar workers tend to be more sedentary, to weigh more, to have higher cholesterol and higher stress levels than blue collar workers [55]. Having said that, we might assume that our results justified this literature, as highly educated people tend to have white collar jobs and, so, they are more prone to work with ICTs, increasing the entrapment feeling and stress levels. Conversely, educational attainment is an important criterion to help low-wage workers to move up the job ladder [56]. Taking into consideration that, besides having higher education, leadership development can lead to better jobs; it is necessary to have people-related competences to exercise the profession focusing on better performances [57], however, the demands of interacting with others may result in emotional exhaustion [58]. Thus, ICTs have an important role for establishing communication between workers and their leader. This dependence of technology to interact with coworkers may justify the higher level of entrapment for this group.

### 4.2. Work Conditions

Having discussed the association between the variables in view of the sociodemographic characteristics, we will now address the impact of the different work conditions (telework, on-site work, not working) on mental health.

#### 4.2.1. Telework Group

Our fourth (H4) hypothesis considered that telework might have negatively impacted mental health and quality of life during the COVID-19 pandemic. Namely, that teleworkers might present higher imagined surveillance, mobile maintenance expectation, communication overload and entrapment, which would increase levels of depression, anxiety and stress and decrease flourishing. To evaluate all the relations between the variables, a medial seriation analysis was conducted linking the work situation to the mental health issues considered (depression, anxiety and stress). First, our findings suggest that telework is related to high imagined surveillance and high communication overload. The high imagined surveillance is directly related to high mobile maintenance expectations, depression, and entrapment. It is important to note that, according to the literature, this association between the usage of mobiles and mental health can be very harmful for people when this addiction goes to another level. As teleworkers intensively use ICTs to work, they spend a long time on their mobile phones, which can lead to further mental health disorders related to mobile addiction, such as nomophobia, i.e., the fear of being far from one’s own phone [59].

Moreover, communication overload is directly related to high stress and entrapment. According to Contreras [60], teleworking demanded the creation of virtual teams, which may include members that are geographically distant, while working together at the same time or asynchronously, creating challenges for the leaders and members to interact with each other, as there might be different time zones or personal time to work. Still according to the authors [60], the leaders of those virtual teams needed competences to keep the team motivated and collaborating, as the actual situation of teleworking may provoke the feeling of isolation and demotivation, affecting the health of workers. Moreover, negative leadership was associated with worse well-being and job satisfaction of the team members, thus, social support was important to avoid emotional exhaustion [61]. All in all, our findings support previous ones, that teleworking demanded more communication through ICTs, and that it may cause a higher pressure to be in touch with coworkers, i.e., communication overload, affecting their mental health.

In addition, high entrapment was directly related to high depression, anxiety, and stress. The literature about technostress discusses the impact of the intense usage of technology in daily activities and health issues. Even though it is still unclear what kind of technostress causes eventual mental disorders, it is a recent field of research; there has been an increasing investment in this area [62]. On the other hand, it is known that technostress is negatively associated with mental health [62] and, assuming that entrapment is also negatively associated with mental health (considering depression, anxiety, and stress), it is possible to verify a correlation between these two concepts. Having said that, the pressure to be in touch with others caused by entrapment may result in technostress, sustaining our finding, but further research is needed to investigate this association.

High depression, anxiety and stress are directly related to low levels of flourishing. According to the literature, positive organizational practices are associated with flourishing at work, which is also related to less turnover intention of the employees, better work performance and better relationships between the teams [63,64]. In other words, our investigation found that flourishing was positively associated with mental health, and that it may directly impact the work situation, contributing to a better organizational environment and, eventually, to reducing worker stress associated with the intention to leave the company, i.e., turnover.

In addition, by comparing the mean scores between groups, we showed that people who worked from home had higher entrapment scores than participants who were in layoff status, unemployed or working in their usual workplace. As previously discussed, the intense use of ICTs during telework results in work intensification and interferes with personal life [17], which sustains our results concerning the pressure to stay connected leading to feelings of entrapment.

Our results about telework and entrapment are in accordance with the literature, but there are also other situations involving on-site workers, unemployed persons and layoff workers that might lead to entrapment [65,66]. It is important to note that unemployment and layoff workers were analyzed together due to the reduced number of unemployed participants. Our fifth (H5) hypothesis predicted that on-site workers, unemployed persons and layoff workers might also have low levels of flourishing and high levels of depression, anxiety, and stress, but not correlated to imagined surveillance, communication overload, entrapment, or mobile maintenance expectations, as they were more exposed to the COVID-19 virus but depended less on ICTs. Our findings showed a different result from what was thought and a distinct model for each group.

#### 4.2.2. On-Site Work Group

According to the literature, people that needed to work on-site during the COVID-19 pandemic feared a possible exposure to the virus at work [65] and felt anxious about being infected by the virus [67]. Our findings suggest that this type of worker has high levels of imagined surveillance, and that this negatively impacts anxiety and stress, but that it is not directly correlated to mobile expectations. Moreover, the high level of communication overload was correlated to high levels of entrapment and diminished flourishing. Entrapment was positively associated with depression, anxiety, and stress, while flourishing was negatively associated with depression and anxiety. Previous literature sustains that the intense use of technology at the workplace may also cause technostress, as communication between employees and companies may be mediated by ICTs [68]. Technology at the workplace may increase the workload, as the quantity of messages and requests may demand a lot of time to catch up, e.g., people can spend hours during the workday checking and cleaning their e-mail inbox [68]. ICTs also cause interruptions during the workday activities, stressing employees by demanding multi-tasking and requiring divided attention, e.g., workers during a meeting may be checking their mobiles to be updated if there are new tasks to be done [68]. In addition, employees working on-site also face the necessity of being connected after work, otherwise they feel at disadvantage at work, which results in work-home interference and stress generated by the technology overload also during personal life [68]. Having said that, we suggest that, differently from teleworkers, the direct negative correlation between communication overload and flourishing occurred because workplace workers may have needed to deal with different channels of communication by ICTs while also having on-site demands at the workplace, which may directly impact their job satisfaction and quality of life [69,70].

Furthermore, this group had a specific behavior linked to imagined surveillance and communication overload. On-site workers with appraised imagined surveillance had more anxiety and stress, while those with appraised communication overload had less flourishing and more anxiety, compared to the other groups. This finding suggests that the intense use of technology to communicate and the worry about coworkers in social media had a more harmful impact on mental health and the quality of life of on-site workers. This harmful impact of appraised imagined surveillance and communication overload on this group of workers is sustained by the stress resulting from: the intense technology use for daily on-site tasks (technostress); the increased demand of work requested though ICTs (workload); the need to multi-task during workday activities, shifting between onsite and digital requests (constant interruptions); and the necessity of also being connected outside the workplace, during private life (work-home interference) [68]. All of this could potentially be intensified due to COVID-19.

#### 4.2.3. Not-Working Group

People who are not working suffer from insecurity and social pressure to change their situation [66]. Our results show that the high level of communication overload is associated with anxiety and stress, while these two mental health issues are negatively associated with flourishing, representing a different model from the other two work conditions discussed before. As they are not currently working, we can assume that they might be using social media and ICTs in an effort to return to work or to look for new job opportunities [71]. Layoff data can be related to job security; the literature has already shown that friendship and alliances inside the workplace and with strategic professionals have positive correlations with job security, i.e., with reduced layoff risks [72]. Having said that, we can assume that workers in layoff positions also have high expectations of relationships with people who can accept them back to work, as social networking positively influences job security [72]. That is, the need to stay in touch with strategic professional colleagues may demand the use of different channels to communicate with them, increasing the communication overload, anxiety, and stress. On the other hand, unemployed people experience social losses as they have diminished social contacts, caused by the lack of interaction with coworkers [73]. With the lack work-related social contacts, unemployed participants may use social media to maintain their relationships and to sustain their necessity to interact with others [73]. Thus, to both groups (unemployed and in layoff), social media and communication platforms might represent a way to make up for the diminished social interaction caused by not being included in the workforce, which may justify the negative correlation with anxiety and stress. It is also important to consider that unemployment seems to be related to unhappiness [74]. In line with this, we show that the participants that were not working presented low flourishing, which, in turn, was associated with high anxiety and stress, as previously demonstrated [75]. In addition, communication overload was negatively correlated to mental health, but there was no correlation with entrapment, as opposed to the other two groups. This profile may be sustained by the necessity of having different channels to interact with people, which negatively impacts the participants’ mental health but without the feeling of being forced to use the ICTs, as there might be no task pressure coming from managers or co-workers due their work conditions, as they are not currently working.

## 5. Conclusions

Having discussed all these topics, it is possible to confirm and go beyond our main hypothesis that telework, being associated to the intense usage of ICTs, causes imagined surveillance and communication overload, impacting mobile maintenance expectations and entrapment, harming mental health and quality of life. In addition, our study found that having a child makes the effects of all those mediations stronger, increasing the dependence with technology and decreasing the mental health and quality of life of teleworkers. In addition, flourishing works as a protector against all the mental health issues in all the analyzed work conditions. Currently, there is still interest for telework even after the pandemic period. However, rules and work regulations to work remotely are needed to protect the worker from exaggerated usage of ICTs, as there might be a lot of interference from work on family and social life [16,76].

In November 2021, a change in the work law concerning the rights of the employee to disconnect from work after the work period, occurred [77]. The right of workers who are parents of children up to 8 years old to maintain telework was also approved [78]. With the eventual end of the pandemic, workers seem to prefer to stay at telework but in a hybrid model, staying at home for some days during the week, taking advantage of such flexibility, and some other days at the office to socialize and use the company infrastructure, bringing together the putative benefits of both worlds [79,80]. These choices should be better discussed, and future directions should be further investigated. This research is not without limitations. Since we recruited participants through social networks such as Facebook, Instagram and WhatsApp, our results might be biased, as those that do not use social networks were not able to access the link to answer the questionnaire. It is possible that this group of people, not contemplated in this study, might either be better at managing work-life balance, opting out on social networks, or even worse, thus, eliminating their online fingerprint in order to manage the stress of digital migration and telework. Moreover, the snowball sampling strategy followed to recruit respondents precludes the generalizability of current findings because the sample was not representative. Future studies should attempt to replicate this mediation hypothesis by recruiting representative samples of the different work situations and in other countries.

Although previous studies suggest an increase in social media use during COVID-19 in students (e.g., [81]), others state that such an increase led to social media fatigue and intention of discontinuation [82] partly due to misinformation sharing [83]. Thus, one possible outcome of the feelings of entrapment and stress due to the pressure of being connected to social networks for work-related purposes, found here, might be that social networks are less used for leisure. Kovacs and collaborators [84] showed that, amid COVID-19, loneliness was not curtailed by social networks, but by face-to face interactions. Vacchiano and Bollano [85] also stated that screen-based leisure activities did not promote relatedness. Hence, if the most impacted people by social network use are not accessing our link, then, our results might be understated.

Moreover, the fact that we have obtained a significantly higher percentage of female respondents might also constitute a limitation since men are underrepresented. This might be related to the fact that women are relationally motivated to participate in social networking, such as maintaining social ties and accessing social information, while men are motivated to gain general information [86]. It is possible that women are more motivated to participate in web surveys compared to men (e.g., [87]) because they are more focused on interpersonal utility than on the personal benefits of information acquisition [88].

Finally, it is important to mention that traditional views on SEM stand for using sample sizes of 10 cases per parameter, which would suggest the need for a larger sample. However, sample size requirements depend on model complexity and on other factors (e.g., normality of the data, missing patterns). In our case, assumptions of data normality were met and there were no missing values. Further, most recent simulations studies recommend rather small sample sizes as enough [89,90,91]. As such, we have estimated the minimum sample size for a desired statistical power level of 0.8, in a model with nine observed variables and a probability level of 0.05. The recommended minimum sample size was 56. After testing our models, we observed that the statistical power of the model for the group with 55 participants was 0.99, and the statistical power for the group of 75 participants was 1.0. Nevertheless, future studies should aim to reproduce these findings using larger samples.

Our data are of paramount importance and suggest that we rethink the generalized dissemination of telework, driven by the necessity of lockdown but potentially gaining post-pandemic ground, given the increase in work intensity and productivity in occupations suitable for working at home during the pandemic [92]. However, these same authors point out that declines in productivity are also observed and strongly associated to declines in mental health and well-being. Moreover, as previously stated, women tend to assume more of the household responsibilities when working from home; there is a need to think about different strategies between genders to mitigate the negative effects of COVID-19 on workers [49]. Here, we substantiated this thesis and recommended a closer look at the specificities of the worker and the creation of guidelines to protect mental health in the telework era.

## Figures and Tables

**Table 1:** Sample Demographic Characteristics.

	Females (n = 409)	Males (n = 91)	Total (n = 500)
**Age**			
Mean	44.6 (SD = 9.7)	44.7 (SD = 9.0)	44.6 (SD = 9.6)
Minimum	20	28	20
Maximum	75	73	75
**Education**			
9 years	6 (1.5%)	4 (4.4%)	10 (2.0%)
12 years	62 (15.2%)	14 (15.4%)	76 (15.2%)
Bachelor	235 (57.5%)	44 (48.4%)	279 (55.8%)
Master	92 (22.5%)	23 (25.3%)	115 (23.0%)
PhD	14 (3.4%)	6 (6.6%)	20 (4.0%)
**Marital Status**			
Single	88 (21.5%)	22 (24.2%)	110 (22.0%)
Married or cohabiting	256 (62.6%)	62 (68.1%)	318 (63.6%)
Divorced	58 (14.2%)	7 (7.7%)	65 (13.0%)
Widower	7 (1.7%)	-	7 (1.4%)
**Number of Children**			
No children	122 (29.8%)	26 (28.6%)	148 (29.6%)
One child	111 (27.1%)	23 (25.3%)	134 (26.8%)
Two children	130 (31.8%)	32 (35.2%)	162 (32.4%)
Three children	37 (9.0%)	8 (.8%)	45 (9.0%)
Four children	6 (1.5%)	2 (2.2%)	8 (1.6%)
Five children	2 (0.5%)	-	2 (0.4%)
Six children	1 (0.2%)	-	1 (0.2%)
**Nationality**			
Angolan	1 (0.2%)	1 (1.1%)	2 (0.4%)
Brazilan	12 (2.9%)	1 (1.1%)	13 (2.6%)
British	-	1 (1.1%)	1 (0.2%)
Dutch	1 (0.2%)	-	1 (0.2%)
French	2 (0.5%)	-	2 (0.4%)
Portuguese	393 (96.1%)	87 (95.6%)	480 (96.0%)
Spanish	-	1 (1.1%)	1 (0.2%)
**Work Regimen**			
Working on behalf of others	310 (75.8%)	68 (74.7%)	378 (75.6%)
Freelancers	78 (19.1%)	22 824.2%)	100 (20.0%)
Student workers	21 (5.1%)	1 (1.1%)	22 (4.4%)
**Working situation**			
Working from home	310 (75.8%)	60 (65.9%)	370 (74.0%)
Usual workplace	35 (8.6%)	20 (22.0%)	55 (11.0%)
Layoff status	46 (11.2%)	7 (7.7%)	53 (10.6%)
Unemployed	18 (4.4%)	4 (4.4%)	22 (4.4%)

**Table 2:** Correlations matrix between entrapment, flourishing, mobile maintenance expectation, depression, anxiety, and stress.

	1	2	3	4	5	6
1. Entrapment	-	−0.10 *	0.42 **	0.33 **	0.33 **	0.38 **
2. Flourishing		-	0.02	−0.38 **	−0.31 **	−0.24 **
3. MME			-	0.17 **	0.20 *	0.16 **
4. Depression				-	0.77 **	0.78 **
5. Anxiety					-	0.79 **
6. Stress						-

** *p* < 0.01; * *p* < 0.05.; MME—Mobile maintenance expectation.

**Table 3:** Tests of Between-Subjects Effects for Work Situation.

Dependent Variables	Mean Square	df	F	*p*	Partial Eta Squared	Observed Power
Entrapment	10.54	2	10.83	0.001	0.04	0.99
Flourishing	1.00	2	1.11	0.33	0.04	0.25
MME	1.59	2	3.41	0.03	0.01	0.64
Depression	0.23	2	0.69	0.50	0.01	0.17
Anxiety	0	2	0.17	0.85	0.01	0.08
Stress	0.13	2	2.08	0.13	0.01	0.43

MME—Mobile maintenance expectation.

## Data Availability

The raw data are available in the Open Science Framework (OSF) repository at DOI: 10.17605/OSF.IO/4H76T and can be accessed via this link https://osf.io/4h76t/?view_only=53bb59cce65f4c728642a8555780ce6f (accessed on 3 January 2022).

## References

[1] Murdock K.K. (2013). Texting while stressed: Implications for students’ burnout, sleep, and well-being. Psychol. Pop. Media Cult..

[2] Benson V., Hand C., Hartshorne R. (2019). How compulsive use of social media affects performance: Insights from the UK by purpose of use. Behav. Inf. Technol..

[3] Wilmer H.H., Sherman L.E., Chein J.M. (2017). Smartphones and cognition: A review of research exploring the links between mobile technology habits and cognitive functioning. Front. Psychol..

[4] Ito M., Kawahara J.-I. (2017). Effect of the presence of a mobile phone during a spatial visual search. Jpn. Psychol. Res..

[5] Ward A.F., Duke K., Gneezy A., Bos M.W. (2017). Brain drain: The mere presence of one’s own smartphone reduces available cognitive capacity. J. Assoc. Consum. Res..

[6] Harpaz I. (2002). Advantages and aisadvantages of telecommuting for the individual, organization and society. Work Study.

[7] Pyöriä P. (2011). Managing telework: Risks, fears and rules. Manag. Res. Rev..

[8] Tavares A.I. (2017). Telework and health effects review. Int. J. Healthc..

[9] Athanasiadou C., Theriou G. (2021). Telework: Systematic Literature Review and Future Research Agenda. Heliyon.

[10] Belzunegui-Eraso A., Erro-Garcés A. (2020). Teleworking in the Context of the COVID-19 Crisis. Sustainability.

[11] Delanoeije J., Verbruggen M. (2020). Between-person and within-person dffects of telework: A quasi-field experiment. Eur. J. Work Organ. Psychol..

[12] Anderson A.J., Kaplan S.A., Vega R.P. (2015). The impact of telework on emotional experience: When, and for whom, does telework improve daily affective well-being?. Eur. J. Work Organ. Psychol..

[13] Gradisar M., Wolfson A.R., Harvey A.G., Hale L., Rosenberg R., Czeisler C.A. (2013). The sleep and technology use of Americans: Findings from the National Sleep Foundation’s 2011 sleep in America poll. J. Clin. Sleep Med..

[14] Rosen L., Carrier L.M., Miller A., Rokkum J., Ruiz A. (2016). Sleeping with technology: Cognitive, affective, and technology usage predictors of sleep problems among college students. Sleep Health.

[15] Upreti A., Musalay P. (2018). Fear of missing out, mobile phone dependency and entrapment in undergraduate students. Applied Psychology Readings.

[16] Maeneja R., Abreu A.M. (2020). The ubiquity of ICTs: Mental health risks reinforced by the COVID-19 crisis. Psicol. Saúde Doenças.

[17] Messenger J.C. (2017). Working anytime, anywhere: The evolution of telework and its effects on the world of work. Int. Labour Off..

[18] Digital Devices Deprive Brain of Needed Downtime. https://www.nytimes.com/2010/08/25/technology/25brain.html?_r=1&th=&emc=th&pagewanted=print.

[19] Aranda J.H., Baig S. (2018). Toward “JOMO”: The joy of missing out and the freedom of disconnecting. Proceedings of the Mobile HCI ’18: 20th International Conference on Human-Computer Interaction with Mobile Devices and Services.

[20] Aitamurto T., Won A.S., Sakshuwong S., Kim B., Sadeghi Y., Stein K., Royal P.G., Kircos C.L. (2021). From FOMO to JOMO: Examining the fear and joy of missing out and presence in a 360° video viewing experience. Proceedings of the CHI ’21: 2021 CHI Conference on Human Factors in Computing Systems.

[21] Duffy B.E., Chan N.K. (2019). “You never really know who’s looking”: Imagined surveillance across social media platforms. New Media Soc..

[22] Yu L., Cao X., Liu Z., Wang J. (2018). Excessive social media use at work: Exploring the effects of social media overload on job performance. Inf. Technol. People.

[23] Elyana A., Ajija S.R., Sridadi A.R., Setyawati A., Emur A.P. (2020). Information Overload and Communication Overload on Social Media Exhaustion and Job Performance. Syst. Rev. Pharm..

[24] Hall J.A., Baym N.K. (2012). Calling and texting (too much): Mobile maintenance expectations, (over)dependence, entrapment, and friendship Satisfaction. New Media Soc..

[25] Li X. (2021). The more the better? A comparative study of the relationships among multimodal connectedness, online communication, and relational outcomes in China and the United States. Chin. J. Commun..

[26] Steele R.G., Hall J.A., Christofferson J.L. (2020). Conceptualizing digital stress in adolescents and young adults: Toward the development of an empirically based model. Clin. Child. Fam. Psychol. Rev..

[27] Carillo K., Cachat-Rosset G., Marsan J., Saba T., Klarsfeld A. (2021). Adjusting to epidemic-induced telework: Empirical insights from teleworkers in France. Eur. J. Inf. Syst..

[28] Boavida N., Moniz A.B. (2020). Virtual work in Portugal: A literature review. Int. J. Work. Cond..

[29] Tavares F., Santos E., Diogo A., Ratten V. (2020). Teleworking in Portuguese communities during the COVID-19 pandemic. J. Enterp. Communities People Places Glob. Econ..

[30] Achdut N., Refaeli T. (2020). Unemployment and psychological distress among young people during the COVID-19 pandemic: Psychological resources and risk factors. Int. J. Environ. Res. Public. Health.

[31] Giorgi G., Lecca L.I., Alessio F., Finstad G.L., Bondanini G., Lulli L.G., Arcangeli G., Mucci N. (2020). COVID-19-related mental health effects in the workplace: A narrative review. Int. J. Environ. Res. Public. Health.

[32] Ferreira L.N., Pereira L.N., da Fé Brás M., Ilchuk K. (2021). Quality of life under the COVID-19 quarantine. Qual. Life Res..

[33] Jacukowicz A., Merecz-Kot D. (2020). Work-related Internet use as a threat to work-life balance—A comparison between the emerging on-line professions and traditional office work. Int. J. Occup. Med. Environ. Health.

[34] Pais-Ribeiro J., Honrado A., Leal I. (2004). Contribution to the adaptation study of the Portuguese adaptation of the Lovibond and Lovibond Depression Anxiety Stress Scales (EADS) with 21 items. Psicol. Saúde Doenças.

[35] Silva A.J., Caetano A. (2013). Validation of the Flourishing Scale and Scale of Positive and Negative Experience in Portugal. Soc. Indic. Res..

[36] Lovibond P.F., Lovibond S.H. (1995). The structure of negative emotional states: Comparison of the Depression Anxiety Stress Scales (DASS) with the Beck Depression and Anxiety Inventories. Behav. Res. Ther..

[37] Diener E., Wirtz D., Tov W., Kim-Prieto C., Choi D., Oishi S., Biswas-Diener R. (2010). New well-being measures: Short scales to assess flourishing and positive and negative feelings. Soc. Indic. Res..

[38] Cohen J. (1988). Statistical Power Analysis for the Social Sciences.

[39] Hayes A.F. (2013). Introduction to mediation, moderation, and conditional process analysis: A regression-based approach. J. Educ. Meas..

[40] Hoyle R.H., Smith G.T. (1994). Formulating clinical research hypotheses as structural equation models: A conceptual overview. J. Consult. Clin. Psychol..

[41] Arbuckle James L. (2012). IBM SPSS Amos 21 User’s Guide.

[42] Preacher K.J., Hayes A.F. (2008). Asymptotic and resampling strategies for assessing and comparing indirect effects in multiple mediator models. Behav. Res. Methods.

[43] Pieh C., Budimir S., Probst T. (2020). The effect of age, gender, income, work, and physical activity on mental health during coronavirus disease (COVID-19) lockdown in Austria. J. Psychosom. Res..

[44] Shahriarirad R., Erfani A., Ranjbar K., Bazrafshan A., Mirahmadizadeh A. (2021). The mental health impact of COVID-19 outbreak: A nationwide survey in Iran. Int. J. Ment. Health Syst..

[45] De Choudhury M., Sharma S.S., Logar T., Eekhout W., Nielsen R.C. (2017). Gender and cross-cultural differences in social media disclosures of mental illness. Proceedings of the CSCW’17: 2017 ACM Conference on Computer Supported Cooperative Work and Social Computing.

[46] Elmer T., Mepham K., Stadtfeld C. (2020). Students under lockdown: Comparisons of students’ social networks and mental health before and during the COVID-19 crisis in Switzerland. PLoS ONE.

[47] Tifferet S. (2020). Gender differences in social support on social network sites: A meta-analysis. Cyberpsychol. Behav. Soc. Netw..

[48] Kneavel M. (2021). Relationship between gender, stress, and quality of social support. Psychol. Rep..

[49] Nguyen M.H., Armoogum J. (2021). Perception and Preference for Home-Based Telework in the COVID-19 Era: A Gender-Based Analysis in Hanoi, Vietnam. Sustainability.

[50] Merkaš M., Perić K., Žulec A. (2021). Parent distraction with technology and child social competence during the COVID-19 pandemic: The role of parental emotional stability. J. Fam. Commun..

[51] Verweij R., Helmerhorst K., Keizer R. (2021). Work-to-family conflict, family-to-work conflict and their relation to perceived parenting and parent-child relationship before and during the COVID-19 lockdown. OSF.

[52] Ugur Z.B. (2020). Does having children bring life satisfaction in Europe?. J. Happiness. Stud..

[53] Bauer N.S. (2018). Technoference over time and parenting. Pediatr. Res..

[54] Chen C., Perry P., Yang Y., Yang C. (2017). Decent work in the Chinese apparel industry: Comparative analysis of blue-collar and white-collar garment workers. Sustainability.

[55] Aginsky K.D., Constantinou D., Delport M., Watson E.D. (2017). Cardiovascular disease risk profile and readiness to change in blue- and white-collar workers. Fam. Community Health.

[56] Abel J.R., Florida R., Gabe T.M. (2018). Can low-wage workers find better jobs?. FRB of New York Staff Report.

[57] Ahmed R., Anantatmula V.S. (2017). Empirical study of project managers leadership competence and project performance. Eng. Manag. J..

[58] Dhamija P., Chiarini A., Shapla S. (2021). Technology and leadership styles: A review of trends between 2003 and 2021. TQM J..

[59] Bhattacharya S., Bashar M.A., Srivastava A., Singh A. (2019). NOMOPHOBIA: NO MObile PHone PhoBIA. J. Fam. Med. Prim. Care.

[60] Contreras F., Baykal E., Abid G. (2020). E-leadership and teleworking in times of COVID-19 and beyond: What we know and where do we go. Front. Psychol..

[61] Dolce V., Vayre E., Molino M., Ghislieri C. (2020). Far away, so close? The role of destructive leadership in the job demands–resources and recovery model in emergency telework. Soc. Sci..

[62] Dragano N., Lunau T. (2020). Technostress at work and mental health: Concepts and research results. Curr. Opin. Psychiatry.

[63] Redelinghuys K., Rothmann S., Botha E. (2019). Flourishing-at-work: The role of positive organizational practices. Psychol. Rep..

[64] Coetzee M., Oosthuizen R.M. (2017). Work-role psychosocial flourishing: Its mediation role on workplace bullying and employee turnover intention. J. Psychol. Afr..

[65] Pamidimukkala A., Kermanshachi S. (2021). Impact of COVID-19 on field and office workforce in construction industry. Proj. Leadersh. Soc..

[66] Rafi M.A., Mamun M.A., Hsan K., Hossain M., Gozal D. (2019). Psychological implications of unemployment among Bangladesh civil service job seekers: A pilot study. Front. Psychiatry..

[67] Eguchi H., Hino A., Inoue A., Tsuji M., Tateishi S., Ando H., Nagata T., Matsuda S., Fujino Y. (2021). Effect of Anxiety About COVID-19 Infection in the Workplace on the Association Between Job Demands and Psychological Distress. Front. Public Health.

[68] Stich J.-F., Tarafdar M., Cooper C.L. (2018). Electronic communication in the workplace: Boon or bane?. J. Organ. Eff. People Perform..

[69] Yin P., Ou C.X.J., Davison R.M., Wu J. (2018). Coping with mobile technology overload in the workplace. Internet Res..

[70] Cho J., Lee H.E., Kim H. (2019). Effects of communication-oriented overload in mobile instant messaging on role stressors, burnout, and turnover intention in the workplace. Int. J. Commun..

[71] Mowbray J.A., Hall H. (2021). Using social media during job search: The case of 16–24-year-olds in Scotland. J. Inf. Sci..

[72] Wu L. (2013). Social network effects on productivity and job security: Evidence from the adoption of a social networking tool. Inf. Syst. Res..

[73] Feuls M., Fieseler C., Suphan A. (2014). A social net? Internet and social media use during unemployment. Work. Employ. Soc..

[74] Sameem S., Buryi P. (2019). Impact of unemployment on happiness in the United States. Appl. Econ. Lett..

[75] Pompili M., Innamorati M., Sampogna G., Albert U., Carmassi C., Carrà G., Cirulli F., Erbuto D., Luciano M., Nanni G. (2022). The impact of COVID-19 on unemployment across Italy: Consequences for those affected by psychiatric conditions. J. Affect. Disord..

[76] Vyas L., Butakhieo N. (2021). The impact of working from home during COVID-19 on work and life domains: An exploratory study on Hong Kong. Policy Des. Pract..

[77] Empresas Obrigadas a Abster-se de Contactar os Trabalhadores no Período de Descanso. https://www.publico.pt/2021/11/03/economia/noticia/empresas-obrigadas-absterse-contactar-trabalhadores-periodo-descanso-1983507.

[78] Aprovado Direito ao Teletrabalho Para Quem Tem Filhos até oito Anos. https://www.publico.pt/2021/11/02/economia/noticia/deputados-aprovam-alargamento-teletrabalho-pais-filhos-ate-8-anos-1983438.

[79] Pataki-Bittó F., Kapusy K. (2021). Work environment transformation in the post COVID-19 based on work values of the future workforce. J. Corp. Real. Estate.

[80] Wang Y., Liu Y., Cui W., Tang J., Zhang H., Walston D., Zhang D. (2021). Returning to the office during the COVID-19 pandemic recovery: Early indicators from China. Proceedings of the CHI EA ’21: Extended Abstracts of the 2021 CHI Conference on Human Factors in Computing Systems.

[81] Gómez-Galán J., Martínez-López J.Á., Lázaro-Pérez C., Sarasola Sánchez-Serrano J.L. (2020). Social networks consumption and addiction in college students during the COVID-19 pandemic: Educational approach to responsible use. Sustainability.

[82] Liu H., Liu W., Yoganathan V., Osburg V.-S. (2021). COVID-19 information overload and generation Z’s social media discontinuance intention during the pandemic lockdown. Technol. Forecast. Soc. Chang..

[83] Islam A.K.M.N., Laato S., Talukder S., Sutinen E. (2020). Misinformation sharing and social media fatigue during COVID-19: An affordance and cognitive load perspective. Technol. Forecast. Soc. Chang..

[84] Kovacs B., Caplan N., Grob S., King M. (2021). Social networks and loneliness during the COVID-19 pandemic. Socius.

[85] Vacchiano M., Bolano D. (2021). Online and offline leisure, relatedness and psychological distress: A study of young people in Switzerland. Leis. Stud..

[86] Krasnova H., Veltri N.F., Eling N., Buxmann P. (2017). Why men and women continue to use social networking sites: The role of gender differences. J. Strateg. Inf. Syst..

[87] Busby D., Yoshida K. (2013). Challenges with online research for couples and families: Evaluating nonrespondents and the differential impact of incentives. J. Child. Fam. Stud..

[88] Kalleitner F., Mühlböck M., Kittel B. (2020). What’s the benefit of a video? The effect of nonmaterial incentives on response rate and bias in web surveys. Soc. Sci. Comput. Rev..

[89] Deng L., Yang M., Marcoulides K.M. (2018). Structural equation modeling with many variables: A systematic review of issues and developments. Front. Psychol..

[90] Sideridis G., Simos P., Papanicolaou A., Fletcher J. (2014). Using structural equation modeling to assess functional connectivity in the brain: Power and sample size considerations. Educ. Psychol. Meas..

[91] Wolf E.J., Harrington K.M., Clark S.L., Miller M.W. (2013). Sample size requirements for structural equation models: An evaluation of power, bias, and solution propriety. Educ. Psychol. Meas..

[92] Etheridge B., Tang L., Wang Y. (2020). Worker productivity during lockdown and working from home: Evidence from self-reports. COVID Econ..

